# Police emergency dispatch model based on emergency level analysis and traffic planning

**DOI:** 10.1371/journal.pone.0348072

**Published:** 2026-07-27

**Authors:** Shi Qiao, Panpan Xu

**Affiliations:** 1 School of Command Tactics, Henan Police College, Zhengzhou, China; 2 School of Economics and Management, Shanghai Maritime University, Shanghai, China; Universiti Sains Malaysia, MALAYSIA

## Abstract

Emergency dispatching and rescue during emergencies have become a hot research topic in the field of transportation planning. Therefore, to address the low efficiency of cross-regional police emergency dispatch, the study carries out relevant exploration on the basis of emergency event level analysis and transportation planning. The emergency rescue demand evaluation system is constructed using the superior and inferior solution distance method to carry out the emergency hierarchy analysis. The police emergency dispatch model is constructed and solved using the improved particle swarm algorithm. The validation revealed that the improved solution algorithm improved the iteration efficiency by 54.33% on average and reduced the iteration time overhead by 56.10% on average over the particle swarm algorithm and the beetle antennae search algorithm. In the scheduling of emergency 1, the material demand satisfaction rate of all demand points was above 80%, among which the vegetable material demand satisfaction rate was 100%. In the scheduling of emergency 2, the demand for supplies at all demand points was above 91.3%, with the demand satisfaction rate for tent supplies as high as 100%. The above results indicate that the police emergency dispatch method can leverage the full advantages of transportation networks. By integrating intelligent optimization algorithms with artificial intelligence technology, the method achieves material dispatch for cross-regional emergency rescue incidents. This method is both feasible and practical.

## 1. Introduction

In recent years, the increasing integration of urban transportation and the intensification of cross-regional socioeconomic activities have made emergency incidents one of the primary threats to public safety and social stability. Emergencies are typically characterized by suddenness, urgency, randomness, destructiveness, and unpredictability. Moreover, once they occur, they often affect multiple administrative regions, requiring rapid and coordinated responses across departments and regions [1]. However, in practice, cross-regional Police Emergency Dispatch (PED) still faces operational bottlenecks and challenges. First, border handoff procedures often constrain the dispatch of vehicles and personnel across administrative boundaries. Convoys must wait or undergo re-coordination at regional checkpoints, which leads to significant delays in response time [2]. Second, the limited number of frontline escort personnel and transport vehicles makes it difficult to achieve rapid and flexible resource allocation when multiple high-priority missions occur simultaneously. In addition, the absence of unified information portals and standardized interfaces between regions hinders real-time data sharing and diminishes the effectiveness of cross-regional coordination. More critically, differences in priority classification and resource allocation rules among jurisdictions result in inconsistent and fragmented emergency resource distribution, which may lead to unmet demands and the loss of optimal rescue opportunities. These issues directly undermine the timeliness and efficiency of emergency response. Moreover, existing dispatch strategies are usually reactive and lack a unified framework for evaluating priorities and a coordinated, multimodal scheduling mechanism.

The objective of this study is to develop and validate a cross-regional PED model that utilizes Technique for Order Preference by Similarity to an Ideal Solution (TOPSIS)-based priorities for routing and allocation via Improved Particle Swarm Optimization (IPSO) to minimize weighted response time and unmet demand. This approach is intended to address the identified problems. The proposed model has clear practical applications, with intended users including Emergency Operation Centers (EOCs), police logistics divisions, and transportation authorities. The PED model enhances the coordination and dispatch efficiency of cross-regional escort convoys by dynamically ranking and scheduling emergency priorities. It also reduces border handoff delays, improves Emergency Supply (ES) satisfaction rates, and optimizes the integrated use of air, rail, and road transportation modes, thereby improving overall response efficiency. These improvements are expected to be reflected in Key Performance Indicators (KPIs) such as average response time, demand satisfaction rate, and total rescue transportation cost.

The remaining parts of the research are organized as follows. The Literature Review Section reviews related studies and highlights the contributions of this work. The Methods and Materials Section presents the Emergency Level Analysis (ELA) method, the PED model framework, and the improved IPSO algorithm. The Results Section reports the results of scenario-based validation. The Discussion and Conclusion Section concludes the research and outlines future research directions.

### Acronyms

**Table:** 

Acronym	Full name	Acronym	Full name
PED	Police Emergency Dispatch	ELA	Emergency Level Analysis
TOPSIS	Technique for Order Preference by Similarity to an Ideal Solution	SPs	Supply Points
IPSO	Improved Particle Swarm Optimization	TFN	Triangular Fuzzy Number
KPIs	Key Performance Indicators	EW	Entropy Weight
ES	Emergency Supply	GCC	Gray Correlation Coefficient
OF	Objective Function	CDP	Collector-Distributor Points
DP	Demand Points	BAS	Beetle Antennae Search
DM	Dispatch Model	HOP	Historical Optimal Position
TP	Traffic Planning	AHP-TOPSIS	Analytic Hierarchy Process-TOPSIS
CRITIC-TOPSIS	CRiteria Importance Through Intercriteria Correlation-TOPSIS	VIKOR	VlseKriterijumska Optimizacija I Kompromisno Resenje
VRP	Vehicle Routing Problem	GA	Genetic Algorithm

## 2 Literature review

### 2.1 Concepts and variables

Emergency dispatch research typically involves several core components. Supply Points (SP) represent the origin of emergency materials, Collector-Distributor Points (CDP) act as transfer hubs, and Demand Points (DPs) represent affected areas. ES refers to materials distributed during emergencies. Decision variables typically describe allocation quantities and transportation flows. KPIs, on the other hand, include response time, supply satisfaction rate, and transportation cost.

### 2.2 Theoretical linkages

Most studies follow a similar theoretical framework in which disaster severity indicators (e.g., casualty levels, infrastructure damage, and material shortages) are assessed first to determine urgency levels. These levels are mapped into priority rankings for dispatch planning. These priority levels guide optimization models that balance efficiency (timeliness and cost) with equity (minimum service guarantees for each DP).

### 2.3 Typical calculation pipelines and algorithm development

Previous studies have developed various approaches to improve emergency Dispatch Models (DMs). In the field of urgent evaluation and DM, Liu and Qian [3] proposed a multi-objective Emergency Services (ESs) distribution model. They did so by specifying objective functions and vehicle constraints. Kergosien et al. [4] applied discrete event simulation to optimize ambulance fleet scheduling. Ding et al. [5] addressed multi-point ES scheduling using interval grey numbers and Genetic Algorithms (GAs). Zhang et al. [6] introduced entropy weight (EW) and dynamic PSO to optimize emergency medical supply distribution. For algorithmic optimization, Wang et al. [7] presented an adaptive weighted differential evolutionary algorithm to improve dispatch global optimality. Yuan and Wang [8] applied super network theory and projection algorithms for chemical park emergencies. Chen et al. [9] used spatiotemporal transformer networks for dynamic prediction regarding advanced artificial intelligence and learning-based methods. Shaik et al. [10] adopted multi-agent reinforcement learning for real-time monitoring. Li et al. [11] proposed ReinforceRouting to handle real-time route changes during disasters.

These studies commonly use Analytic Hierarchy Process-TOPSIS (AHP-TOPSIS), CRiteria Importance Through Intercriteria Correlation-TOPSIS (CRITIC-TOPSIS), or VlseKriterijumska Optimizacija I Kompromisno Resenje (VIKOR) for urgency assessment [12,13]. They usually use normalization and closeness coefficients to quantify severity levels, as well as metaheuristics or deep learning methods to improve optimization and real-time decision-making. In general, existing research mainly focuses on prioritizing emergncy needs through multi-indicator decision-making methods and improving resource scheduling efficiency using optimization algorithms. However, most of this research concentrates on emergency resource scheduling problems in single-region or localized disaster scenarios, typically assuming that supply nodes, transit nodes, and demand nodes are located within the same administrative region and coordinated by a unified scheduling entity. Research on emergency scheduling problems involving coordination across multiple administrative regions, complex transportation networks, and cross-regional resource allocation remains relatively limited.

### 2.4 Research gap and contributions

Although existing research offers valuable insights into emergency dispatching, most studies are confined to a single region. Cross-regional scheduling is still in the conceptual stage. Quantitative modeling and algorithmic optimization for large-scale, multi-node, multi-modal networks are still scarce. Moreover, priority mapping mechanisms between urgency evaluation and dispatch are often absent or simplistic. This study closes these gaps by combining TOPSIS-based ELA and an enhanced IPSO algorithm to develop a cross-regional PED model. The study focuses on enhancing scheduling efficiency and rescue effectiveness within multi-regional, multi-modal transportation networks.

## 3 Methods and materials

The study first conducts an ELA and uses TOPSIS to build an analytical model to estimate the urgency of the rescue demand, to address the low PED efficiency for cross-regional transportation in emergency situations. On this basis, PED model construction for cross-regional TP is carried out, and the PSO algorithm is improved to solve the model using IPSO algorithm.

The data used in this study are obtained from publicly available sources or authorized emergency drill materials. They do not contain personal privacy or sensitive information and have been anonymized and desensitized. The data complies with scientific research ethics and relevant management requirements. No human subjects or animal experiments are involved, and no additional ethical approval is required.

### 3.1 Modeling and operational assumptions

**Fig 1:**
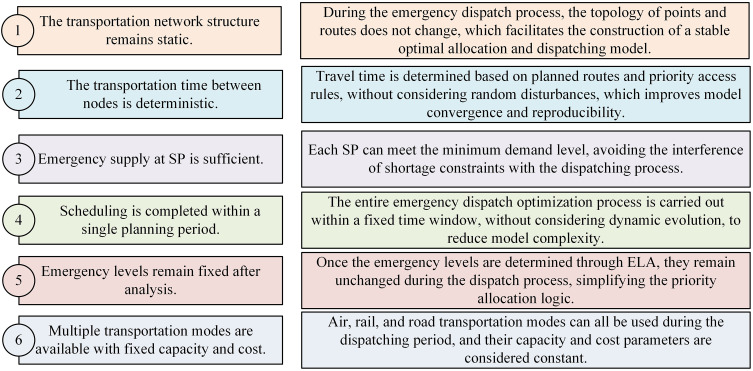
Model-related assumptions. The model assumes that: (1) the transportation network structure remains static; (2) travel time between nodes is deterministic; (3) emergency supplies at each supply point satisfy the minimum demand; (4) scheduling is completed within a single planning period; (5) emergency levels remain unchanged after analysis; and (6) multiple transportation modes are available with fixed capacities and costs. SP denotes supply point.

To ensure the model’s solubility and computational efficiency, as well as its alignment with the operational mechanisms of cross-regional emergency dispatch, several basic assumptions are made during the construction and simulation processes, as illustrated in Fig 1.

In Fig 1, the assumption of a static transport network structure provides a stable optimization foundation for the overall model framework and path planning. The assumption of deterministic transport time is reflected in parameter setting and scheduling optimization. The assumption of sufficient material reserves at SPs is used in constraint modeling to prevent shortages from interfering with the scheduling process. Single-planning-cycle scheduling is assumed in model solving and simulation. A fixed urgency level is assumed throughout the ELA and priority mapping process. Furthermore, the assumption of multiple transport modes available with fixed capacities and costs supports transport mode selection and resource allocation, enabling the coordinated optimization of multiple transport modes.

### 3.2 TOPSIS-based emergency level analysis

**Fig 2:**
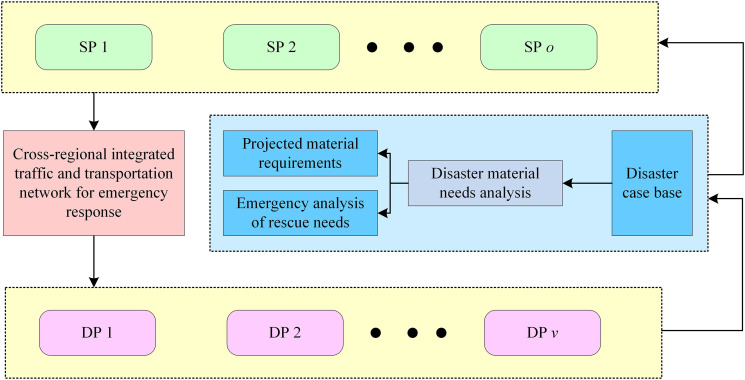
Cross-regional ELA process. Disaster cases are analyzed to estimate emergency material demand and support allocation from supply points to demand points. SP and DP denote supply point and demand point, respectively.

In cross-regional emergency rescue, the imbalance between the DPs and rescue supplies leads to low rescue efficiency [14]. To maximize the effectiveness of rescue, the study firstly conducted ELA. In the context of emergency rescue occurrence, information communication and transportation are the key facilities for rescue. Therefore, the study mainly focuses on the related departments to conduct ELA. The specific process is shown in Fig 2.

**Fig 3:**
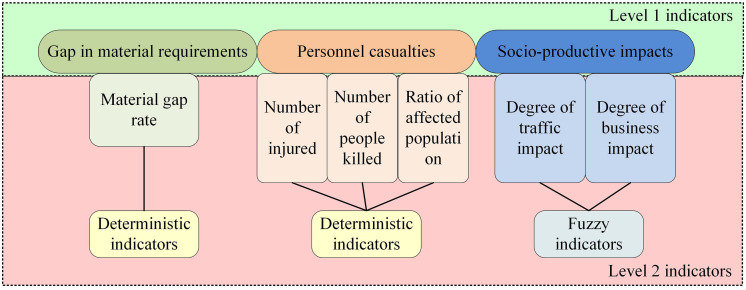
Evaluation system of emergency rescue demand level. The system includes material gaps, personnel casualties, and socio-productive impacts, evaluated using deterministic and fuzzy indicators.

In Fig 2, o denotes the SP. v denotes the DP. The overall analysis process of this study includes data collection and processing, ELA, priority mapping, DM construction, solver configuration with baseline settings, and result validation. Among these steps, ELA serves as the core component for achieving cross-regional emergency dispatch by comprehensively assessing the urgency level of DPs through multiple indicators. The determination of urgency is mainly based on the material shortage at the rescue site, the number of casualties and the proportion of the affected population, as well as the economic losses caused to social production activities. Material shortages are the key factor driving cross-regional rescue. Casualties directly reflect the threat emergencies pose to human life and safety. The impact on social production represents the disruption to economic activities [15]. Meanwhile, the study mainly assesses the social production functioning from two levels: transportation and business transit. Therefore, the evaluation system of rescue demand level under emergency is shown in Fig 3.

From Fig 3, the study divides the hierarchical evaluation system into two levels. The first-level indicators are the material demand gap, human casualties and the impact on social production, as proposed earlier. The material shortage rate is the second-level indicator that corresponds to the gap in the material demand, and the number of injuries, deaths, and the ratio of the affected population to the total population comprise the second-level indicator that corresponds to the casualty situation. The secondary indicators corresponding to the first-level indicator of social production impact are transportation impact and enterprise impact. Traffic impact and enterprise impact are fuzzy indicators, and the study introduces Triangular Fuzzy Number (TFN) to calculate them. TFN offers significant advantages when it comes to handling ambiguity and uncertainty. It can accurately represent data that is difficult to characterize with exact numerical values. Its membership function adopts a commonly used standard form, as shown in Equation (1) [16].


$$\begin{array}{c} b_{a}=\left\{ \begin{array}{c} @l@0,c<a_{\text{1}}\\ \frac{c-a_{\text{1}}}{a_{\text{2}}-a_{\text{1}}},a_{\text{1}}\leq c\leq a_{\text{2}}\\ \frac{a_{\text{3}}-c}{a_{\text{3}}-a_{\text{2}}},a_{\text{2}}\leq c\leq a_{\text{3}}\\ 0,c>a_{\text{3}} \end{array}\right. \end{array}$$ (1)

In Equation (1), a denotes a fuzzy number. c denotes an evaluation index. $b_{a}$ denotes the affiliation function of fuzzy number a. $a_{\text{1}}$, $a_{\text{2}}$ and $a_{\text{3}}$ denote the most pessimistic, probable and optimistic number, respectively. When the three are equal, the fuzzy number a is real. In the TFN, the degree of impact of transportation and business is categorized into five linguistic variables, namely, “very severe”, “severe”, “fair”, “mild”, and “very mild”. The five linguistic variables correspond to the TFN numbers (0.7,1.0,1.0), (0.5,0.7,0.9), (0.3,0.5,0.7), (0.1,0.3,0.5), and (0.0,0.0,0.3), respectively. This study uses the TFN values of five linguistic variables to parameterize the corresponding variable weights at their maximum possible values. The method estimates the most probable value from the observations, as shown in Equation (2) [17].


$$\begin{array}{c} \left\{ \begin{array}{c} @l@a=\left\{a_{\text{1}},a_{\text{2}},a_{\text{3}}\right\}=\omega_{\text{1}}a_{\text{1}}+\omega_{\text{2}}a_{\text{2}}+\omega_{\text{3}}a_{\text{3}}\\ \omega_{\text{1}}=\omega_{\text{3}}=\frac{\text{1}}{\text{6}}\\ \omega_{\text{2}}=\frac{\text{4}}{\text{6}} \end{array}\right. \end{array}$$ (2)

In Equation (2), $\omega_{\text{1}}$, $\omega_{\text{2}}$, and $\omega_{\text{3}}$ denote the weights corresponding to TFN numbers $a_{\text{1}}$, $a_{\text{2}}$, and $a_{\text{3}}$, respectively. The five linguistic variable weights are further derived from Equation (2) as 0.95, 0.7, 0.5, 0.3, and 0.05, respectively. Multiple attribute decision-making studies have clearly identified language variables and their TFN weights [18]. These values are widely applicable in contexts such as disaster management and emergency logistics. The study also assesses the impact on transportation and commerce. The same classification system is highly appropriate. The study introduces the TOPSIS method of ELA to construct a rescue demand level analysis model. ELA and TOPSIS can achieve efficient and transparent urgency quantification and priority sorting using multiple indicators [19,20]. In the cross-regional emergency rescue evaluation process, the first relevant rescue strategy is made by the relevant organizations such as the emergency department in the event of emergencies to the urgency of the rescue needs of the affected location. Therefore, the initial rescue decision matrix expression is shown in Equation (3).


$$\begin{array}{c} 𝐀=\left[\begin{array}{cccc} @cccc@a_{11} & a_{12} & \cdots & a_{1n}\\ a_{21} & a_{22} & \cdots & a_{2n}\\ \vdots & \vdots & \cdots & \vdots \\ a_{\delta 1} & a_{\delta 2} & \cdots & a_{\delta n} \end{array}\right] \end{array}$$ (3)

In Equation (3), 𝐀 denotes the initial decision matrix. $a_{\delta n}$ denotes the fuzzy number of qualitative indicators. δ denotes the material DPs, and n denotes the evaluation indicators. The qualitative indicators are processed using TFN to further obtain a clear 𝐀. Meanwhile, 𝐀 is normalized, and the final 𝐀 is updated, as shown in Equation (4).


$$\begin{array}{c} 𝐁=\left[\begin{array}{cccc} @cccc@b_{11} & b_{12} & \cdots & b_{1n}\\ b_{21} & b_{22} & \cdots & b_{2n}\\ \vdots & \vdots & \cdots & \vdots \\ b_{\delta 1} & b_{\delta 2} & \cdots & b_{\delta n} \end{array}\right] \end{array}$$ (4)

In Equation (4), 𝐁 denotes 𝐀 after normalization, $b_{\delta n}$ denotes fuzzy number after normalization, and $b_{\delta n}=\mathrm{\backslash raisebox}1ex\backslash (a_{\delta n}\backslash )\;/\;\mathrm{\backslash raisebox}\mathrm{-1ex}\backslash (maxa_{\delta n}\backslash )$. The study calculates each afflicted point’s objective weight using the EW approach since the disaster information that each DP transmits during an emergency is unique, as shown in Equation (5).


$$\begin{array}{c} \left\{ \begin{array}{c} @l@C_{ij}=\frac{b_{ij}}{\sum_{i}^{\delta }b_{ij}}\\ e_{j}=-\mathrm{\backslash raisebox}1ex\backslash (1\backslash )\;/\;\mathrm{\backslash raisebox}\mathrm{-1ex}\backslash (ln\delta \backslash )\sum_{i}^{\delta }C_{ij}lnC_{ij}\\ d_{j}=\frac{1-e_{j}}{n-\sum_{j}^{n}e_{j}} \end{array}\right. \end{array}$$ (5)

In Equation (5), $C_{ij}$ is the contribution of the j th indicator to the i th DP, where i∈δ, j∈n. $e_{j}$ is the information entropy of the j th indicator. $d_{j}$ is the weight of the j th indicator. Simultaneously, the EW approach and hierarchical analysis are combined to calculate the overall weight of the indicators, as shown in Equation (6).


$$\begin{array}{c} \omega_{j}=\lambda \partial_{j}+(1-\lambda )d_{j} \end{array}$$ (6)

In Equation (6), $\omega_{j}$ is the combined weight of the j th indicator. λ is the preference coefficient, calculated using the analytic hierarchy process. The study draws on existing research experience in the field of emergency dispatch and sets it at 0.5 [21,22]. According to the determined weights of each influencing factor in the decision-making judgment, the weighted normalization matrix can be further obtained, as shown in Equation (7).


$$\begin{array}{c} 𝐂=\left[\begin{array}{cccc} @cccc@c_{11} & c_{12} & \cdots & c_{1n}\\ c_{21} & c_{22} & \cdots & c_{2n}\\ \vdots & \vdots & \cdots & \vdots \\ c_{\delta 1} & c_{\delta 2} & \cdots & c_{\delta n} \end{array}\right] \end{array}$$ (7)

In Equation (7), 𝐂 denotes the weighted normalization matrix and $c_{\delta n}$ denotes the reference sample vector. The maximum and minimum values in the reference sample constitute the positive and negative ideal solutions of the evaluation indexes. The specific expression is shown in Equation (8).


$$\begin{array}{c} \left\{ \begin{array}{c} @l@{𝐂}^{+}=\left(c_{\text{1}}^{+},c_{\text{2}}^{+},\cdots c_{n}^{+}\right)c_{j}^{+}=maxc_{ij}\\ {𝐂}^{-}=\left(c_{\text{1}}^{-},c_{\text{2}}^{-},\cdots c_{n}^{-}\right)c_{j}^{-}=minc_{ij} \end{array}\right. \end{array}$$ (8)

In Equation (8), ${𝐂}^{+}$ and ${𝐂}^{-}$ denote the positive and negative ideal solutions, respectively. On this basis, the matrix of Gray Correlation Coefficient (GCC) is calculated using gray correlation theory to calculate the GCC and correlation degree of DPs and positive ideal solutions. In this case, the matrix of GCC expression is shown in Equation (9).


$$\begin{array}{c} {𝐅}^{+}=\left[\begin{array}{cccc} @cccc@f_{11}^{+} & f_{12}^{+} & \cdots & f_{1n}^{+}\\ f_{21}^{+} & f_{22}^{+} & \cdots & f_{2n}^{+}\\ \vdots & \vdots & \cdots & \vdots \\ f_{\delta 1}^{+} & f_{\delta 2}^{+} & \cdots & f_{\delta n}^{+} \end{array}\right] \end{array}$$ (9)

In Equation (9), ${𝐅}^{+}$ represents the matrix of strength and closeness coefficients used to quantitatively describe and compare the relationship between factors in one system or between two systems. $f_{\delta n}^{+}$ denotes the coefficient of strength and closeness used to quantitatively describe and compare the relationship between factors in one system or between two systems. The formula for calculating $f_{\delta n}^{+}$ and correlation between a DP and the corresponding indicator is shown in Equation (10).


$$\begin{array}{c} \left\{ \begin{array}{c} @l@f_{ij}^{+}=\frac{\underset{i}{min}\underset{j}{min}\left|c^{+}-c_{ij}\right|+\kappa \cdot \underset{i}{max}\underset{j}{max}\left|c^{+}-c_{ij}\right|}{\left|c^{+}-c_{ij}\right|+\kappa \cdot \underset{i}{max}\underset{j}{max}\left|c^{+}-c_{ij}\right|}\\ r_{i}^{+}=\frac{\text{1}}{n}\sum_{j}f_{ij}^{+} \end{array}\right. \end{array}$$ (10)

In Equation (10), $f_{ij}^{+}$ is the GCC of the j th indicator. $r_{i}^{+}$ is the positive ideal gray correlation. κ is the resolution coefficient, which takes the value of 0.5. The relative closeness of the gray correlation of the DP is shown in Equation (11).


$$\begin{array}{c} R=\frac{r_{i}^{+}}{r_{i}^{+}+r_{i}^{-}} \end{array}$$ (11)

In Equation (11), R denotes the relative closeness of the gray correlation. $r_{i}^{-}$ denotes negative ideal gray correlation. The implementation steps of ELA based on the TOPSIS method are shown in Table 1.

**Table 1:** Pseudo-code of TOPSIS-based ELA.

Procedure ELA_TOPSIS_TFN_EW_GCC(X, TFN_map, α = 0.5, ζ = 0.5):
# Step 1: Convert qualitative indicators to crisp values
For each qualitative indicator j:
For each DP i:
(lij, mij, uij) ← TFN_map[Xij]
Xij ← Defuzzify_TFN(lij, mij, uij) # e.g., (l + 4m + u)/6
# Step 2: Normalize indicators
For each indicator j:
If j is benefit-type:
rij ← Xij / sqrt(Σ_i Xij^2)
Else:
rij ← (min_i Xij) / Xij
# Step 3: Entropy weight calculation
pj(i) ← rij / (Σ_i rij + ε)
Ej ← -k * Σ_i [pj(i) * ln(pj(i) + ε)] # k = 1/ln(m)
w_EW(j) ← (1 – Ej) / Σ_j (1 – Ej)
# Step 4: AHP weight calculation
w_AHP(j) ← AHP_weight(j)
# Step 5: Combined weights
w(j) ← α * w_AHP(j) + (1 – α) * w_EW(j)
# Step 6: Weighted normalized decision matrix
vij ← w(j) * rij
# Step 7: Ideal solutions
vj+ ← max_i vij
vj- ← min_i vij
# Step 8: Grey correlation coefficient
Δij+ ← |vij – vj + |
Δmin ← min_{i,j} Δij+
Δmax ← max_{i,j} Δij+
γij ← (Δmin + ζ*Δmax) / (Δij+ + ζ*Δmax)
# Step 9: Closeness coefficient
Ci ← Σ_j [γij * w(j)] / Σ_j w(j)
# Step 10: Ranking
π ← argsort(Ci, descending = True)
return C, π
End Procedure

### 3.3. PED model based on emergency level analysis and transportation planning

**Fig 4:**
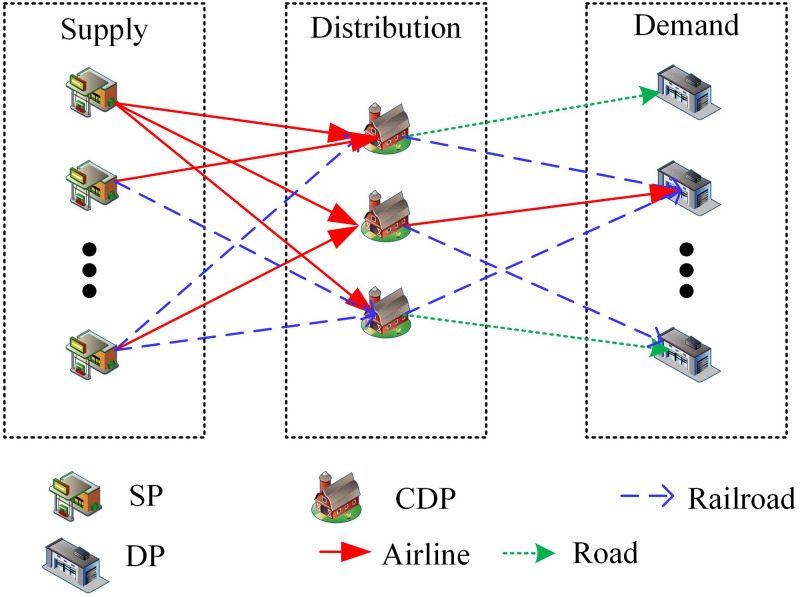
Interregional dispatch network structure based on emergency hierarchy and transportation planning. Supply, transfer, and demand points are connected through differentiated dispatch routes, with colored arrows indicating different emergency levels and transportation schemes.

Based on the ELA approach in the previous section, the study further combines TP for PED model construction. PED model is an optimisation framework for the allocation of police resources in emergency situations. The model comprehensively considers the urgency level of the incident, traffic conditions in the target area, and resource availability to construct a dispatch priority function, and on this basis, it realises resource path optimisation and minimisation of response time. The PED model is designed by leveraging artificial intelligence technology. Transportation and distribution of materials are the key aspects of emergency rescue. Therefore, the PED model is mainly designed around ES in emergency. Since emergency dispatching includes multiple materials, multiple origins and destinations, and multiple transports in cross-regional emergency rescue [23,24], the study describes it as a network topology, as shown in Fig 4.

**Fig 5:**
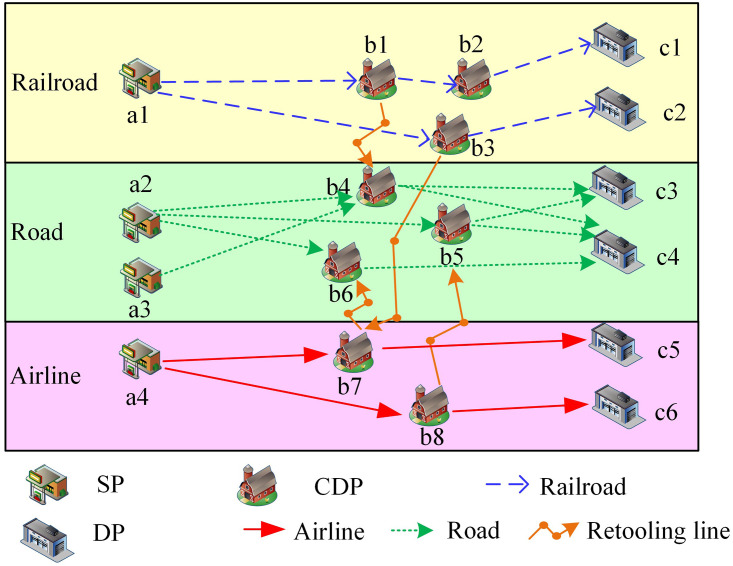
Transportation planning network structure. Supply, transfer, and demand points are connected by railway, road, and airline routes, with orange links indicating intermodal transfers. a, b, and c denote SP, CDP, and DP, respectively.

In Fig 4, the study divides the scheduling process into a three-level network, in which the first level is mainly responsible for supplying the materials needed at the DPs, which are produced and raised by the relevant departments, enterprises, etc., and transported to the material CDPs through a variety of means of transportation. The second-level network is the material CDPs, which are mainly responsible for receiving and transferring the materials transported by the SPs and distributing them to the sites in need of materials. The tertiary network is the DPs, which are mainly responsible for receiving materials from the while network and then distributing the materials in the vicinity through helicopters and land transportation [25,26]. Meanwhile, the study divides the network for transportation dispatched across the region, as shown in Fig 5.

In Fig 5, the study divides the transportation network into three layers: railroad, road, and airline. The material CDP in each layer should have the following principles: convenient transportation, perfect scale of equipment and facilities, high social impact situation and advantageous natural conditions. Combined with the above, the PED model is shown in Equation (12).


$$\begin{array}{c} P=\left\{ \begin{array}{c} @l@minf_{\text{1}}=\sum_{l}\sum_{o}\sum_{k}x_{ol}^{km}\cdot U_{ol}^{km}\cdot t_{ol}^{km}+\sum_{k}\sum_{l}U_{ol}^{km}\cdot t_{l}^{k}+\sum_{l}\sum_{v}\sum_{k}x_{lv}^{km}\cdot U_{lv}^{km}\cdot t_{lv}^{km}\\ minf_{\text{2}}=\sum_{l}\sum_{o}\sum_{k}x_{ol}^{km}\cdot U_{ol}^{km}\cdot q_{ol}^{km}\cdot h_{ol}^{km}+\sum_{k}\sum_{l}U_{il}^{km}\cdot h_{l}^{k}+\sum_{l}\sum_{v}\sum_{k}x_{lv}^{km}\cdot U_{lv}^{km}\cdot q_{lv}^{km}\cdot h_{lv}^{km}\\ minf_{\text{3}}=\sum_{v}\sum_{k}U_{v}^{k}\cdot \alpha_{v}^{k}\cdot \beta_{v}^{k} \end{array}\right. \end{array}$$ (12)

In Equation (12), P denotes the PED model. $f_{\text{1}}$ denotes the timeliness OF of rescue and $f_{\text{2}}$ denotes the economy OF of rescue. $f_{\text{3}}$ denotes the utility OF of rescue. l denotes a CDP. k denotes a certain type of supplies. m denotes the number of planned routes for transportation of supplies. $x_{ol}^{km}$ denotes the transportation of relief supplies from o to l, and takes a value equal to 1 when the transportation strategy chosen by k is the m strategy, or 0 when the opposite is applied. $U_{ol}^{km}$ denotes the transportation volume of $x_{ol}^{km}$, and $t_{ol}^{km}$ denotes the time overhead spent on transportation by $x_{ol}^{km}$. $t_{l}^{k}$ denotes the time overhead spent on distribution and transshipment of supplies at l. $x_{lv}^{km}$ denotes the transportation of relief supplies from l to v. $U_{lv}^{km}$ denotes the transportation volume of $x_{lv}^{km}$, and $t_{lv}^{km}$ denotes the transportation time of $x_{lv}^{km}$. $q_{ol}^{km}$ and $h_{ol}^{km}$ denote the distance and cost of transportation for the m transportation method planned from o to l, respectively. $h_{l}^{k}$ denotes the unit transit cost of material k except at CDP l. $q_{lv}^{km}$ and $h_{lv}^{km}$ denote the transportation mileage and transportation cost of the m kind of transportation method selected by material k from CDP l to DP v, respectively. $U_{v}^{k}$ denotes the lack of quantity of the material at the DP. $\beta_{v}^{k}$ denotes the emergency relief demand level of v after the occurrence of emergencies. $\alpha_{v}^{k}$ denotes the correction factor that sets the premium or discount cost for the benefit of relief material k to the ER. The urgency level obtained from ELA is normalized and incorporated into the correction factor in Equation (12), which serves as the priority weight for each DP. Higher urgency levels lead to higher correction factors, thereby increasing their weight in the allocation objective. The study focuses on the determination of the OF of the PED model in terms of timeliness of rescue, economy of rescue and utility of rescue. Consequently, the study imposes pertinent constraints on the PED model. Less material than the amount of reserve at the SP must be carried from the SP to the CDP. In a similar vein, less material must be provided to the DP from the CDP than must be requested at the CDP. Additionally, the amount of goods conveyed at the CDP cannot be greater than the amount of stuff requested at the CDP. The specific expression is shown in Equation (13).


$$\begin{array}{c} \left\{ \begin{array}{c} @l@U_{ol}^{km}\leq Q_{o}^{k}\\ U_{lv}^{km}\leq E_{l}^{k}\\ \sum_{l}U_{lv}^{km}\leq D_{v}^{k} \end{array}\right. \end{array}$$ (13)

In Equation (13), $Q_{o}^{k}$ denotes the SP’s reserve on material k. $E_{l}^{k}$ denotes the total amount of materiel k accepted by the CDP. $D_{v}^{k}$ denotes the amount of demand for material k at the DP. To ensure the fairness of ER, it is necessary to ensure that the amount of material transported to the disaster site meets the minimum level of material demand service. Furthermore, the original storage quantity of the material at the CDP and the quantity transported to the CDP by the SP should be equal to the quantity of the substance delivered to the DP. The specific expression is shown in Equation (14).


$$\begin{array}{c} \left\{ \begin{array}{c} @l@\sum_{l}U_{lv}^{km}\geq D_{v}^{k}\chi_{o}\\ \sum_{i}U_{ol}^{km}+Q_{l}^{k}=E_{l}^{k}=\sum_{j}U_{lv}^{km} \end{array}\right. \end{array}$$ (14)

In Equation (14), $Q_{l}^{k}$ denotes the reserve at the CDP with respect to material k, and $\chi_{o}$ denotes the minimum demand rate. The sum of the transportation volume of the CDP should be lower than the throughput capacity of the CDP, and the number of transportation means should be lower than the transportation reserve capacity. The specific expression is shown in Equation (15).


$$\begin{array}{c} \left\{ \begin{array}{c} @l@\sum_{k}E_{l}^{k}\leq E_{l}\\ s_{ol}^{km}=\left|\frac{U_{ol}^{km}\cdot \phi_{k}}{W_{k}^{m}}\right|\leq s_{ol}^{m}\\ s_{lv}^{km}=\left|\frac{U_{lv}^{km}\cdot \phi_{k}}{W_{k}^{m}}\right|\leq s_{lv}^{m}\\ U_{v}^{k}=D_{v}^{k}-\sum_{m}\sum_{l}U_{lv}^{km} \end{array}\right. \end{array}$$ (15)

In Equation (15), $E_{l}$ denotes the throughput capacity of the CDP, and $s_{ol}^{km}$ denotes the number of means of transportation of $x_{ol}^{km}$. $s_{lv}^{km}$ denotes the number of transportation means of $x_{lv}^{km}$. $s_{ol}^{m}$ denote the reserved transportation capacity from SP to CDP, and $s_{lv}^{m}$ denote the reserved transportation capacity from CDP to DP. $W_{k}^{m}$ represents the unit carrying capacity of transportation mode m. Transportation capacity is determined by the number of transportation vehicles and their unit carrying capacity. Since different transportation modes have different carrying capacity parameters $W_{k}^{m}$, this model can describe emergency dispatching problems under heterogeneous transportation resources. To effectively solve the model, the study first performs multi-objective processing on the model, determines the priority of the OF $f_{\text{3}}$ according to the principle of human-centeredness, and takes it as the constraint of the model. It then performs weighted single-objective processing. Compared to methods based on the Pareto frontier, the weighted summation method is more computationally efficient and better suited to the requirements of solution speed and decision certainty in emergency scheduling problems. The processing formula is shown in Equation (16) [27].


$$\begin{array}{c} minz=\mu_{\text{1}}\cdot f_{\text{1}}+\mu_{\text{2}}\cdot f_{\text{2}} \end{array}$$ (16)

In Equation (16), $\mu_{\text{1}}$ and $\mu_{\text{2}}$ denote the different weight coefficients of the OF, and z denotes the optimal solution. According to Equation (16), the study utilizes the IPSO algorithm to compute the better solution of the model. The IPSO algorithm is obtained by introducing the Beetle Antennae Search (BAS) algorithm for optimization based on the PSO algorithm [28,29]. The BAS algorithm can improve the optimization rate of the PSO algorithm during the goal search process, increasing the efficiency with which the algorithm finds an optimal solution [30–32]. In the IPSO algorithm, the PED model is mainly transported from two routes (SP-CDPs, and CDPs-DPs) and provides corresponding transportation strategies for various types of materials. It is assumed that there are two supplies to be transported and five sites (a1, a2, a3, a4, and a5) capable of supplying both. There are 2 sites (b1 and b2) that distribute the supplies. There are 4 sites (c1, c2, c3, and c4) that need to receive supplies. The specific particle codes for the two phases are specified in Table 2.

**Table 2:** Example of IPSO algorithm particle encoding.

Particle coding for SP-CDP					CDP-DP particle coding				
–	b1	b2	b1	b2	–	c1	c2	c3	c4
a1	p1	p6	p11	p16	b1	p21	p22	p23	p24
a2	p2	p7	p12	p17	b2	p25	p26	p27	p28
a3	p3	p8	p13	p18	b1	p29	p30	p31	p32
a4	p4	p9	p14	p19	b2	p33	p34	p35	p36
a5	p5	p10	p15	p20	–	–	–	–	–

**Fig 6:**
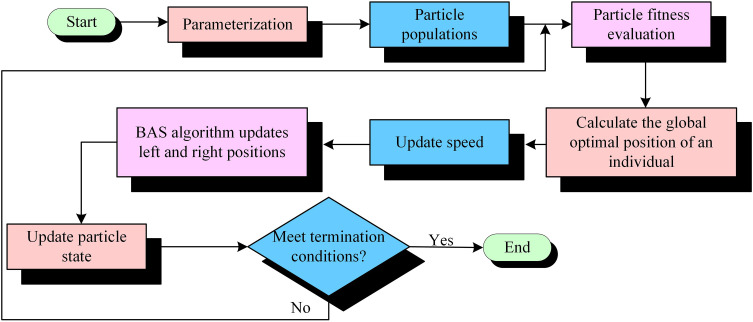
IPSO algorithm solving process in PED model. The algorithm iteratively evaluates particle fitness, updates the global optimum, velocity, position, and particle state until the termination condition is satisfied. BAS denotes beetle antennae search, and IPSO denotes improved particle swarm optimization.

In Table 2, the SP-CDP transportation scheme is 20-dimensional. The CDP-DP transportation scheme is 16-dimensional. p denotes the random number carrying the transportation volume and transportation mode, which takes the value range of [0,1]. Therefore, Fig 6 depicts the IPSO algorithm’s solution flow in accordance with the particle coding approach.

In Fig 6, the IPSO algorithm first sets the relevant parameters to set the specific information about the size of the particle swarm at the very beginning, the update speed and the position [33,34]. The adaptation degree is calculated according to the adaptation function, which is shown in Equation (17).


$$\begin{array}{c} \left\{ \begin{array}{c} @l@Fit=\mu_{\text{1}}\frac{f_{\text{1}}-minf_{\text{1}}}{minf_{\text{1}}}+\mu_{\text{2}}\frac{f_{\text{2}}-minf_{\text{2}}}{minf_{\text{2}}}\\ \mu_{\text{1}}+\mu_{\text{2}}=1 \end{array}\right. \end{array}$$ (17)

In Equation (17), Fit denotes the fitness function. According to the particle adaptation degree, the positions of individual particles and the global optimum are determined, and the particle positions are updated according to the BAS algorithm. The specific update is shown in Equation (18).


$$\begin{array}{c} \left\{ \begin{array}{c} \;\;\;V(t+1)=g_{\text{2}}r_{\text{2}}\left(y^{\prime }(t)-x\left(t\right)\right)+\varepsilon V(t+1)+g_{\text{1}}r_{\text{1}}\left(y(t)-x\left(t\right)\right)\\ \;\;\;x(t+1)=\left\{ \begin{array}{cc} \;\;\;x\left(t\right)+V(t+1) & f\left(x\left(t+1\right)\right)\leq f\left(x{\left(t+1\right)}^{!}\right)\\ \;\;\;x\left(t\right)-V(t+1) & f\left(x\left(t+1\right)\right)>f\left(x{\left(t+1\right)}^{!}\right) \end{array}\right. \end{array}\right. \end{array}$$ (18)

In Equation (18), V(t+1) is the ion update velocity and x(t+1) is the particle update position. x(t) represents the particle position. ε is the inertia factor, and $g_{\text{1}}$ and $g_{\text{2}}$ are the particle movement acceleration. $r_{\text{1}}$ and $r_{\text{2}}$ are uniformly distributed random numbers with values in the range of [0,1]. y(t) is the Historical Optimal Position (HOP) of a single particle. $y^{\prime }\left(t\right)$ is the HOP of all particles of the population. f(x(t+1)) and $f(x(t+1{)}^{!})$ are the target values of left whisker and right whisker respectively. To ascertain whether the maximum iteration has been achieved, the particle state is updated at the end. The scheduling scheme’s output will be executed if the condition is met. Otherwise the adaptation will be brought in with new particles to recalculate until the condition is satisfied and the optimal scheduling scheme is output. To enhance the global search capability of PSOA and avoid premature convergence, this paper constructs a collaborative mechanism between BASA and IPSOA. In each iteration, the position and velocity of the particles are first updated using standard PSOA. The BASA tentacles search mechanism is introduced to evaluate the objective function of the particle’s current position neighborhood and determine the search direction based on the comparison results, thereby enhancing the local search capability. IPSOA introduces a conditional triggering mechanism: when the global optimal solution is not updated in K consecutive iterations, the particle swarm is considered to have entered a stagnant state. At this time, BASA is triggered to perform local perturbation to escape the local optimum and improve the diversity of solutions. The parameter K is used to balance the relationship between global search and local search. When K is too small, local search is triggered frequently, while when K is too large, it will weaken the role of local search. Therefore, the parameter is set to 10 through preliminary experiments. Overall, BASA provides a neighborhood search supplement to PSOA, and the two achieve collaborative updates through Equation (18). Based on the above, the pseudo-code of the PED model implementation is shown in Table 3.

**Table 3:** Pseudo-code of PED model.

Procedure IPSO_PED_Solver(Data, PSO_params, BAS_params, β):
# Step 0: Initialize feasible population
P ← InitializeFeasiblePopulation(N, Data)
For each particle p in P:
p.v ← 0
p.f ← EvaluateObjective(p, Data, β)
p.pbest ← p
p.f_pbest ← p.f
gbest ← argmin_p p.f
stall ← 0
For t = 1 .. T:
# Step 1: Standard PSO update
For each particle p in P:
r1, r2 ~ U(0,1)
p.v ← ω*p.v + c1*r1*(p.pbest – p.pos) + c2*r2*(gbest.pos – p.pos)
p.pos ← ProjectToFeasible(p.pos + p.v, Data)
p.f ← EvaluateObjective(p, Data, β)
If p.f < p.f_pbest:
p.pbest ← p
p.f_pbest ← p.f
# Step 2: BAS neighborhood search
If gbest not improved:
stall ← stall + 1
Else:
stall ← 0
If stall ≥ K:
For each selected particle q:
d ← RandomDirection(unit_length = True)
q_left ← ProjectToFeasible(q.pos + s*d, Data)
q_right ← ProjectToFeasible(q.pos – s*d, Data)
f_left ← EvaluateObjective(q_left, Data, β)
f_right ← EvaluateObjective(q_right, Data, β)
If f_left < f_right:
q.pos ← q_left; q.f ← f_left
Else:
q.pos ← q_right; q.f ← f_right
stall ← 0
# Step 3: Update global best
h ← argmin_p p.f
If h.f < gbest.f:
gbest ← h
# Step 4: Termination check
If Converged(gbest, t): break
return gbest.pos, gbest.f
End Procedure

## 4. Results

To verify the effectiveness of the PED method using the emergence class and TP proposed in this study, PSO algorithm and BAS algorithm are first presented and their solutions to the PED model are compared with IPSO algorithm. Secondly, the scheduling method validation is carried out with two different emergency instances.

### 4.1. Experimental design and data sources

The two typical case studies correspond to the large-scale material dispatch scenario of the 2019 COVID-19 pandemic (instance 1) and the emergency rescue scenario following the 2008 Wenchuan earthquake (instance 2). Data sources include emergency drills, historical public data, transportation information platforms, and supplementary simulation-based data based on characteristics of real-world scenarios. The data is a mix of real-world and simulated data. Real-world data is used to simulate typical cross-regional emergency dispatch scenarios for major public health events and earthquakes. Simulated data supplements the scale and complexity of nodes. Simulated data is generated based on real data through random perturbation and parameter expansion, so that the expanded data maintains similar statistical characteristics to the original data in terms of demand scale and transportation distance. Data collection primarily focuses on emergency transportation records during the 2019–2020 pandemic and public case data from the 2008 Wenchuan earthquake rescue. Data collection methods include systematic data collection, historical data collation, and simulation expansion. Data is used in the experiments after passing scientific research project registration and open data authorization. All data is standardized before using, including distance matrix normalization, transportation time standardization, and material demand smoothing.

In terms of the feasibility of transportation methods, the transportation routes for all scenarios are set based on the actual transportation network or post-disaster transportation recovery capabilities: In the epidemic scenario, the focus is on the regular allocation of materials by railways and highways and emergency air transport channels. In the earthquake scenario, blocked roads and interrupted lines are taken into consideration. Routes that cannot be used are closed or assigned high penalty costs.

### 4.2. Validation of PED instance 1

To verify the effectiveness of the proposed PED model based on ELA and TP for transportation scheduling in emergency, the study firstly validates the results of performance comparison between artificial intelligence optimization algorithms PSO, BAS, and the proposed IPSO in solving the PED model. Based on the typicality and operability principles of PED, the transportation and material hub areas most affected by the epidemic in instance 1 are selected. A public health event at a location with five SPs (a1, a2, a3, a4, and a5), two CDPs (b1 and b2), and four DPs (c1, c2, c3, and c4) are set up. ES is medical protective gear and vegetables. The combination ratio of protective gear is protective clothing: goggles: mask = 1:1:2. The weight of one protective clothing is 0.5 kg, the weight of goggles is 0.03 kg, and the weight of mask is 0.02 kg. The weight of 10,000 sets of medical protective gear is 5.7 t. The set of material transportation modes include air, rail and road. Specific transportation-related information is shown in Table 4.

**Table 4:** Materiel transportation information (mileage, time).

–	b1 (km, h)			b2 (km, h)		
	Airline	Railroad	Road	Airline	Railroad	Road
a1	(826.3, 1.4)	(944.4, 12.6)	(951.0, 9.5)	(856.3, 1.4)	(1021.9, 13.6)	(982.3, 9.8)
a2	652.2, 1.1)	(1024.2, 13.6)	(780.4, 7.8)	(708.0, 1.2)	(1102.4, 14.7)	(896.4, 9.0)
a3	(983.0, 1.6)	(1450.1, 19.3)	(1149.8, 11.5)	(1036.4, 1.7)	(1432.2 19.1)	(1137.3, 11.4)
a4	(869.1, 1.5)	(1310.8, 17.5)	(1043.3, 10.4)	(905.4, 1.5)	–	(1187.0, 11.9)
a5	(1046.1, 1.7)	(1322.4, 17.6)	(1197.0, 12.0)	(1058.4, 1.8)	–	(1236.3, 12.4)
b1	–	–	–	(64.2, 0.3)	(78.0, 1.0)	(87.0, 0.9)
b2	(64.3, 0.3)	(78.4, 1.0)	(88.3, 1.9)	–	–	–
c1	(48.9, 0.2)	(90.0, 1.2)	(68.1, 0.7)	(106.4, 0.5)	(170.0, 2.3)	(150.2, 1.5)
c2	(113.0, 0.6)	(124.3, 1.7)	(156.9, 1.6)	(171.8, 0.9)	(175.1, 2.3)	(243.8, 2.4)
c3	(69.0, 0.3)	(64.8, 0.9)	(74.8, 0.8)	–	–	(14.3, 0.1)
c4	(83.8, 0.4)	(105.4, 1.4)	(103.3, 1.0)	(28.3, 0.1)	(27.3, 0.4)	(36.0, 0.4)

**Fig 7:**
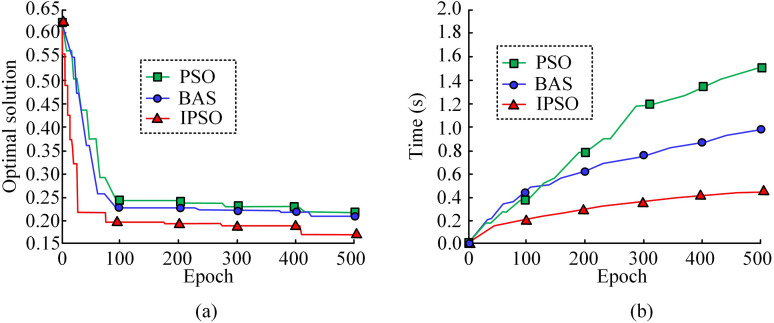
Comparison of the convergence effect of the three algorithms. (a) Comparison of iterative convergence curves of three algorithms. (b) Comparison of the iteration time curves of the three algorithms. PSO, BAS, and IPSO denote particle swarm optimization, beetle antennae search, and improved particle swarm optimization, respectively.

In Table 4, the study sets the average speed of air transportation as 600 km/h, the average speed of railroad as 75 km/h, and the average speed of road as 100 km/h. By programming in Matlab r2019b software, the particles are set to be 100, and the number of iteration of the three algorithms is 500. The final obtained iteration curves of the three algorithms for solving the PED model are compared. The results are shown in Fig 7.

**Fig 8:**
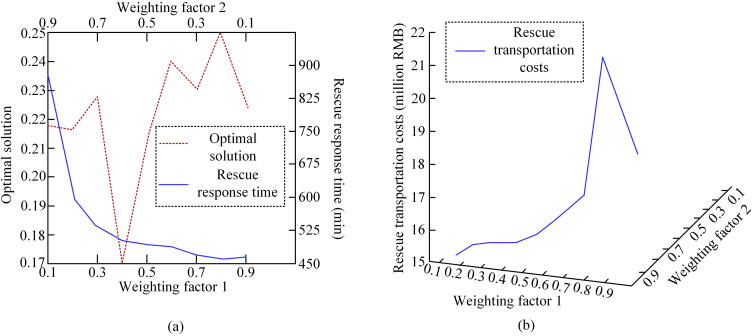
IPSOA solution results. (a) Rescue response time solution results with different weights. (b) Rescue transportation cost solution results with different weights.

Fig 7(a) displays the iterative convergence curves of the three algorithms. The updated IPSO algorithm achieves a lower optimal solution value of 0.17 and converges more quickly. The iterative convergence time of the three methods is shown in Fig 7(b). Each algorithm’s time overhead rises with the number of rounds. The PSO algorithm takes the most time, followed by the BAS algorithm. This indicates that the study to improve the PSO algorithm using the BAS algorithm is reliable and reasonable, and the improved IPSO algorithm is more superior in convergence speed as well as model solving. The algorithm converges quickly in the initial stage. This is mainly because the constraints on transportation capacity, demand, and transportation network structure in the model impose certain limitations on the feasible solution space, enabling the algorithm to quickly approach the optimal solution within a relatively small number of iterations. Simultaneously, IPSOA introduces the BAS local search mechanism on top of particle swarm optimization, which enhances the solution space exploration capability and reduces the risk of getting trapped in local optima. Therefore, this rapid convergence process mainly stems from the combined effect of model constraints and the algorithm’s search mechanism, rather than premature convergence. The scheduling results under different weight coefficients of the PED model obtained by solving according to the IPSO algorithm are shown in Fig 8.

From the optimal value and rescue response time obtained by solving under different weighting parameters in Fig 8(a), the PED model solves the smallest optimal value of 0.17, and the smallest rescue response time of 459.73 min. Combined with the results of solving the rescue transportation cost in Fig 8(b), the IPSO algorithm solves the smallest rescue transportation cost of Rmb14.86 million. As the rescue time decreases, the rescue transportation cost increases. This indicates that the decision maker can choose the appropriate weighting system for the determination of the scheduling scheme at different rescue stages. The study also calculates the urgency score $\beta_{v}^{k}$ of each DP using the TOPSIS comprehensive evaluation model. Then, it normalized the $\beta_{v}^{k}$ to obtain the priority weight of each DP and introduced this weight as a node-level parameter into the PED scheduling model. By converting urgency scores into priority weights through a linear mapping method, consistency between urgency evaluation results and scheduling models is maintained, and the relative urgency of different demand nodes is reflected in the scheduling optimization process. The details are shown in Table 5.

**Table 5:** ELA results and priority mapping.

DP	$\beta_{v}^{k}$	Priority weight	Allocated material quantity (t)	Satisfaction rate (%)
c1	0.93	0.91	10.50	99.87
c2	0.74	0.70	8.28	80.00
c3	0.88	0.85	9.40	96.14
c4	0.95	0.93	11.00	99.72

**Fig 9:**
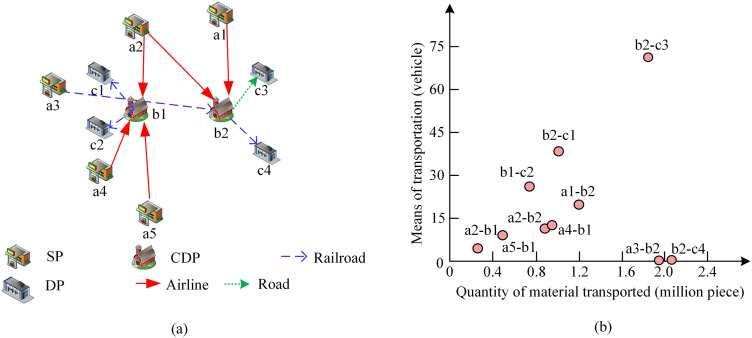
Medical protective gear dispatch program for relief supplies. (a) Scheduling results for medical protective gear. (b) Schematic diagram of the quantity of material transported versus the two-dimensional coordinates of the means of transport.

Table 5 showed that c1 and c4 had the highest ELA urgency scores and corresponding priority weights, resulting in relatively high resource allocation and satisfaction rates during the scheduling process. In contrast, c2 had a priority weight of 0.70, but its satisfaction rate was 80%. This result is obtained by optimizing the overall objective function of the PED model using the IPSO algorithm, taking into account factors such as transportation capacity constraints, supply node inventory limitations, and transportation path costs. The model achieves global optimum by optimizing overall response time and transportation costs while ensuring that each demand node meets the minimum service level constraint, reflecting the trade-off between efficiency and fairness in emergency scheduling during resource allocation. Therefore, based on the above solution results, the study further obtains the optimal scheduling scheme based on the material transportation and the transportation means, as shown in Fig 9.

**Fig 10:**
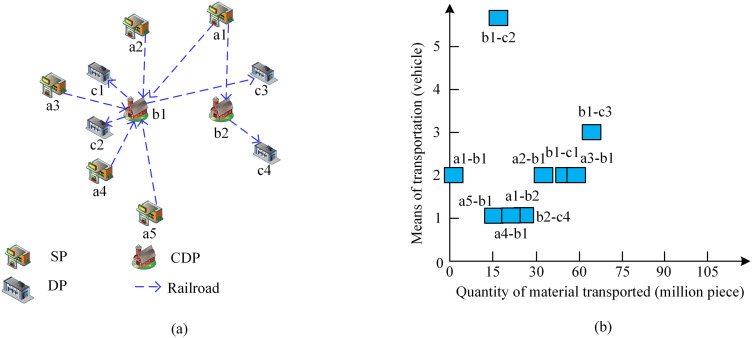
Vegetable movement plan for rescue supplies. (a) Dispatch results for vegetables. (b) Schematic diagram of the quantity of material transported versus the two-dimensional coordinates of the means of transport.

In Fig 9(a), in the PED model solved by IPSO algorithm, the optimal SP to CDP scheduling schemes are a1-b2, a2-b1, a4-b1, and a5-b1 for air transportation of supplies, and a3-b2 for rail transportation of supplies. In addition, in CDP to DP of medical protective gear scheduling, the optimal scheme is b1-c1, b1-c2, b2-c4 for railroad transportation of materials, b2-c3 for road transportation of materials. The results of the two-dimensional coordinate system, as depicted in Fig 9(b), indicate that the number of required air, railroad, and road means of transportation is 56, 65, and 73, respectively. The scheduling scheme for the rescue materials and vegetables is shown in Fig 10.

**Fig 11:**
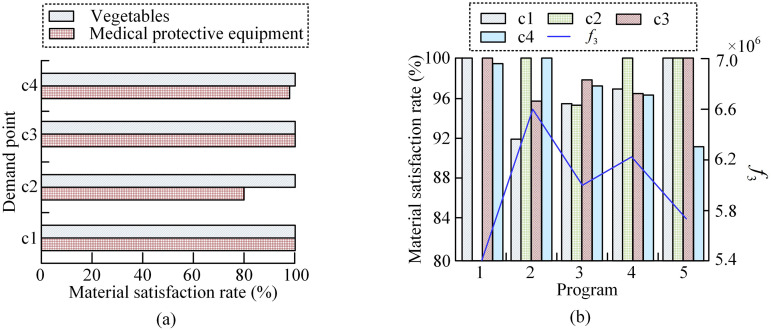
Change in material requirements satisfaction rate and OF under the movement control program. (a) Point-of-need material satisfaction rate under the movement control programme. (b) Variation of *f*_3_ under different scenarios of medical protective gears.

In Fig 10(a), in the PED model, the transportation means for scheduling relief materials and vegetables are mainly railroads. Combined with Fig 10(b) the transportation means required for each planning dispatch and the quantity of transported materials, in larger distances or requiring larger materials, railroad transportation with low transportation cost and large transportation quantity is more favored by DM. According to this scheduling scheme, the study further analyzes the changes in the $f_{\text{3}}$ when adjusting the quantity of materials at the four DPs for the two materials to meet the demand rate and the quantity of materials at the DPs under the situation of constant supply of materials, as shown in Fig 11.

In Fig 11(a), the medical protective gear and vegetable scheduling scheme obtained from the PED model. All four DPs have a 100% material satisfaction rate for vegetables. The material satisfaction rate for medical protective gear is 80% for DP c2 and 99.74% for DP c4. This is mainly due to the fact that the current model does not set clear priority strategies for resource allocation. All types of demand are optimized uniformly according to the objective function, resulting in some delays in the response to medical supplies during certain periods. Vegetables have relatively small demand quantities, more concentrated DP, and lower transportation weights, so they are given priority. Combining the five scheduling scenarios of medical protective gear in Fig 11(b), the minimum material satisfaction rate of all scenarios is 80% and above, which meets the actual rescue needs. In addition, from the changes of the $f_{\text{3}}$ under the five scenarios, $f_{\text{3}}$ under scenario 1 takes the smallest value. When combined with the rescue response time and transportation cost under the different weight parameters of the OF from the previous section, it can be concluded that the rescue loss is lowest when the OF is small. Therefore, it is feasible and reasonable to study the PED of this emergency according to scheme 1.

### 4.3. Validation of PED instance 2

**Fig 12:**
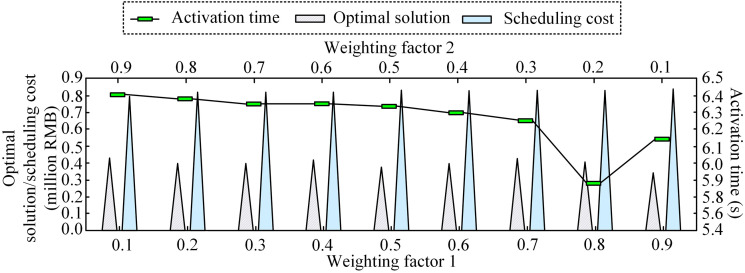
Calculation results of scheduling with different weight values. The bars represent the optimal solution and scheduling cost, while the green markers indicate activation time.

To further verify the effectiveness of the proposed PED model scheduling method, experiments are conducted using a strong earthquake event as an example. The most representative disaster-stricken points and emergency material nodes for post-disaster emergency rescue are selected. Six SPs (a1, a2, a3, a4, a5, and a6), two CDPs (b1 and b2), and five DPs (c1, c2, c3, c4, and c5) are set. The required relief materials are tents and drinking bottled water. The results of scheduling calculations under different weight values are shown in Fig 12.

**Fig 13:**
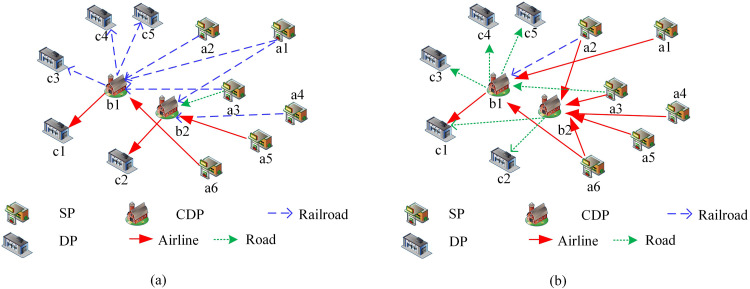
Tent and drinking water dispatch program for relief supplies. (a) Results of the movement of tents for relief supplies. (b) Results of the movement of relief supplies of drink water.

In Fig 12, the scheduling transportation time is inversely proportional to the transportation cost for different values of weights. This indicates that the weight setting sensitivity of the proposed PED model is more desirable. Based on the above results, the IPSO algorithm is further utilized to solve the PED model, and the obtained scheduling scheme is shown in Fig 13.

**Fig 14:**
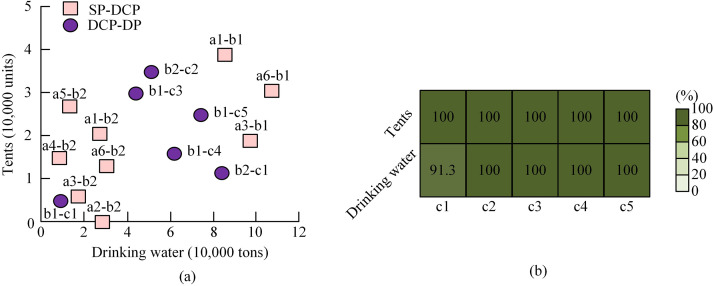
Quantity of material transported and rate of demand met. (a) Quantity of relief supplies dispatched. (b) Material satisfaction rate.

In Fig 13(a), in the scheduling scheme of relief material tents, the transportation modes from the SP to the CDP are mostly railroad transportation and air transportation, while the road transportation is less. This may be because the PED model model takes into account that the example is an earthquake event and the efficiency of road transportation is slow. Combined with the transportation distribution of rescue material drinking water in Fig 13(b), the effectiveness of the proposed PED method can be observed. Therefore, the study further analyzes the quantity of material transported and the demand satisfaction rate under this scheduling scheme, as shown in Fig 14.

Fig 14(a) shows the results of scheduling the quantity of relief supplies at SP-CDP and CDP-DP. The horizontal coordinate indicates the quantity of drinking water and the vertical coordinate indicates the quantity of tents. Combined with the demand fulfillment rate of the two kinds of materials in Fig 14(b), all the supplied quantities of rescue materials tents exceed the actual demanded quantities, and therefore the demands of all demanded sites are fulfilled. The minimum demand fulfillment rate for all DPs in the dispatch result of rescue supplies drinking water is 91.3%. This may be due to the urgency evaluation index of each DP resulting in a lower supply of potable water at DP c1. However, overall, the PED model scheduling method satisfies the minimum demand satisfaction rate, and the scheduling scheme is feasible and reasonable.

### 4.4. Comprehensive validation and comparative analysis

Based on the aforementioned dataset, this study analyzes the stability and feasibility of the proposed PED model. To reduce the influence of randomness and ensure statistical significance, each set of experiments is independently repeated 30 times. The mean and 95% confidence interval are calculated based on the experimental results. The specific results are shown in Table 6.

**Table 6:** Parameter sensitivity verification.

Parameter settings	TOPSIS weight	λ	Number of particles	Response time (h)	Cost (×10^4^ yuan)	Satisfaction rate (%)	Feasibility audit (Yes/No)		
							Supply and demand balance	Throughput constraint	Service level
Baseline	0.50	0.60	100	5.32 ± 0.21	43.17 ± 2.31	98.73 ± 0.48	Yes	Yes	Yes
λ +10%	0.50	0.66	100	5.41 ± 0.25	44.06 ± 2.54	98.12 ± 0.56	Yes	Yes	Yes
TOPSIS+10%	0.55	0.60	100	5.36 ± 0.19	43.42 ± 2.38	98.51 ± 0.52	Yes	Yes	Yes
Number of particles +20	0.50	0.60	120	5.28 ± 0.23	43.01 ± 2.26	98.94 ± 0.44	Yes	Yes	Yes

Table 6 shows that increasing the number of particles slightly decreases the model’s response time and improves the satisfaction rate. Minimal overall performance fluctuations result from adjusting the TOPSIS weights and values, which has a minimal impact on the cost and satisfaction rate. This demonstrates that the proposed PED model is stable under parameter variations and exhibits strong robustness. Furthermore, the supply-demand balance, throughput constraints, and service-level constraints are met under all perturbation scenarios. No resource allocation imbalances or constraint violations are observed. These results further validate the solution’s feasibility and robustness. To verify the optimization capability of the IPSO algorithm in the path planning subproblem, experiments were conducted using a standard VRP benchmark instance with 50 nodes selected from the standard benchmark library for the Vehicle Routing Problem (VRP). Since the standard VRP instance does not contain node priority information, this experiment is only used to evaluate path optimization performance. Under the same parameters and constraints, the results were compared with those of the GA, the greedy strategy, and the deep reinforcement learning-based path planning method (DRL-based routing). The results are shown in Table 7.

**Table 7:** Performance comparison of algorithms on the VRP benchmark dataset.

Method	Response time (h)	Cost (×10^4^ yuan)	Satisfaction rate (%)
IPSO	3.84 ± 0.17	21.63 ± 1.92	99.16 ± 0.41
GA	4.09 ± 0.23	23.27 ± 2.04	96.95 ± 0.52
Greedy strategy	4.63 ± 0.28	25.95 ± 2.34	93.57 ± 0.69
DRL-based routing	3.98 ± 0.21	22.48 ± 1.86	97.83 ± 0.47

In Table 7, the proposed method achieved a response time of 3.84 h on the VRP benchmark dataset, which was 6.12%, 17.06%, and 3.52% shorter than that of GA, the greedy strategy, and DRL-based routing, respectively. The proposed method also reduced transportation costs by 7.05%, 16.67%, and 3.78% compared to the other three methods. Furthermore, the confidence interval of the proposed model exhibits a narrow fluctuation range, indicating its enhanced stability and robustness. Finally, the study further verified the scalability of the PED emergency scheduling model in large-scale networks. Experiments were conducted with scheduling networks of 100, 200, 300, and 500 nodes, respectively, and the IPSO algorithm was used for solution. The details are shown in Table 8.

**Table 8:** Scalability test of the PED model on large-scale networks.

Network size (nodes)	Response time (h)	Cost (×10⁴ yuan)	Satisfaction rate (%)	Average runtime (s)
100	4.27	24.51	98.74	6.92
200	5.12	27.48	97.92	11.86
300	5.96	30.15	96.83	18.43
500	7.48	34.72	95.41	31.27

As shown in Table 8, as the network size gradually increased from 100 nodes to 500 nodes, the model’s response time and transportation costs increased, but the growth trend remained stable. Meanwhile, the average satisfaction rate of each demand node remained at a high level, indicating that the proposed PED scheduling model can maintain stable scheduling performance even as the network size expands. These results demonstrate that the proposed method has good scalability and application potential in large-scale cross-regional emergency dispatch networks.

This study analyzes the time complexity of the PSO, BAS, and IPSO algorithms. The number of particles is N, the problem dimension is D, and the maximum number of iterations is T. The time complexity of the standard PSO algorithm is O(NDT), and the time complexity of the BAS algorithm is O(DT). The proposed IPSO introduces the BAS local search mechanism into the PSO framework, and its overall time complexity is approximately O(NDT), which is on the same order of magnitude as the PSO algorithm, thus not increasing the computational complexity.

## 5. Discussion and conclusion

The study’s key contributions included the development of an emergency level assessment and priority emergency dispatch optimization model. This model integrated urgency analysis, transportation priority, artificial intelligence technology, and an IPSO algorithm combined with the beetle antennae search algorithm. The integration was to enhance the efficiency of emergency rescue in cross-regional integrated transportation networks. In PED case 1, the vegetable supply satisfaction rate at all DPs reached 100%, the minimum satisfaction rate for medical protective equipment was 80%, and the average satisfaction rate was 94.94%. In case 2, the tent supply satisfaction rate reached 100%, the average satisfaction rate for drinking water was 98.26%, and the minimum satisfaction rate was 91.30%. On the standard vehicle routing problem benchmark dataset, the response time of the proposed method was reduced by 6.12% and 17.06% compared with the GA and the greedy strategy, and transportation costs were reduced by 7.05% and 16.67%, respectively. Compared to typical algorithms and benchmark methods, the proposed model achieved a more efficient scheduling response and balanced resource allocation [28,29,32].

Theoretically, this study incorporates a dynamic mapping mechanism of urgency and priority into a cross-regional dispatch optimization framework. It also introduces a measurable priority dispatch factor and formalizes equity requirements as model constraints. This enriches the theoretical system of emergency logistics DM. Unlike traditional models, which focus primarily on time or cost, this study balances scheduling efficiency and fairness in resource allocation. This provides a framework that can be used to solve large-scale emergency transportation problems. At the practical level, this model can directly provide auxiliary decision support for actual management entities such as EOCs, public security traffic management departments, and transportation bureaus. For example, in scenarios involving cross-regional allocation of emergency supplies during public health emergencies or disasters, different SPs, CDPs, and DPs need to quickly formulate scheduling plans. Through priority-driven resource allocation and dynamic constraint scheduling, the proposed method can shorten cross-regional material allocation time, reduce transportation costs, and improve the coverage of each demand node. Compared with existing scheduling methods based on PSO or GA, the proposed PED-IPSO model demonstrates superior overall performance in response time and transportation costs, thus providing more efficient decision support for emergency management departments.

Several limitations remain in the modeling process. The main assumptions include deterministic transportation time, static network structure, split deliveries, and fixed fleet capacity. These assumptions are adopted to simplify the computational complexity of the model and ensure tractable optimization in emergency scenarios where rapid decision-making is required. However, they limit the applicability and external validity of the model in highly dynamic, large-scale scenarios. Future work will address these limitations by introducing dynamic and robust routing mechanisms to handle uncertainty and integrate real-time data assimilation to enhance adaptive responses.

Additionally, the scale and heterogeneity of multi-region experiments will be expanded, and governance and handoff constraints will be incorporated to improve applicability in multi-agency coordinated dispatch. Although case studies based on epidemic and earthquake scenarios have demonstrated the effectiveness, the scope of these studies remains limited. They do not represent all emergency dispatch situations. Future research will focus on larger, more complex, and more diverse scenarios to enhance the model’s external validity and generalizability. Future research will introduce demand node priority labels on the existing VRP benchmark dataset to construct an extended test set that better reflects the characteristics of emergency dispatch. Simultaneously, combining traffic simulation tools with digital twin systems can provide real-time perception of road network conditions, thereby achieving dynamic dispatch. Furthermore, cross-regional government collaboration rules will be quantified into model constraints to enhance the model’s practical application value in complex emergency management scenarios.

### Symbols

**Table:** 

Symbol	Definition	Symbol	Definition
o	SP	v	DP
a	Fuzzy number	$b_{a}$	Affiliation function of fuzzy number a
c	Evaluation index	$a_{\text{1}}$,$a_{\text{2}}$ and $a_{\text{3}}$	Most pessimistic, probable and optimistic number, respectively
$\omega_{\text{1}}$, $\omega_{\text{2}}$ and $\omega_{\text{3}}$	Weights corresponding to TFN numbers $a_{\text{1}}$, $a_{\text{2}}$ and $a_{\text{3}}$, respectively	𝐀	Initial decision matrix
$a_{\delta n}$	Fuzzy number of qualitative indicators	δ	Material DPs
n	Evaluation indicators	𝐁	𝐀 after normalization
$b_{\delta n}$	Fuzzy number after normalization	$C_{ij}$	Contribution of the j th indicator to the i th DP, where i∈m, j∈n
$e_{j}$	Information entropy of the j th indicator	$d_{j}$	Weight of the j th indicator
$\omega_{j}$	Combined weight of the j th indicator	λ	Preference coefficient
𝐂	Weighted normalization matrix	$c_{\delta n}$	Reference sample vector
${𝐂}^{+}$ and ${𝐂}^{-}$	Positive and negative ideal solutions	${𝐅}^{+}$	Matrix of strength and closeness coefficients
$f_{\delta n}^{+}$	Coefficient of strength and closeness	$f_{ij}^{+}$	GCC of the j th indicator
$r_{i}^{+}$	Positive ideal gray correlation	κ	Resolution coefficient
R	Relative closeness of the gray correlation	$r_{i}^{-}$	Negative ideal gray correlation
P	PED model	$f_{\text{1}}$, $f_{\text{2}}$, and $f_{\text{3}}$	Timeliness, economy and utility OFs
l	CDP	k	Certain type of supplies
m	Number of planned routes for transportation of supplies	$x_{ol}^{km}$, $x_{lv}^{km}$	Transportation of relief supplies from o to l, l to v
$U_{ol}^{km}$, $U_{lv}^{km}$	Transportation volume of $x_{ol}^{km}$, $x_{lv}^{km}$	$t_{ol}^{km}$, $t_{lv}^{km}$	Time overhead spent on transportation by $x_{ol}^{km}$, $x_{lv}^{km}$
$q_{lv}^{km}$ and $h_{lv}^{km}$	Transportation mileage and transportation cost	$U_{v}^{k}$	The lack of quantity of the material at the DP
$q_{ol}^{km}$ and $h_{ol}^{km}$	Distance and cost of transportation	$h_{l}^{k}$	Unit transit cost of material k except at CDP
$\beta_{v}^{k}$	Emergency relief demand level of v after the occurrence of emergencies	$\alpha_{v}^{k}$	Correction factor that sets the premium or discount cost for the benefit of relief material k to the ER
$Q_{o}^{k}$	The SP’s reserve on material k	$E_{l}^{k}$	The total amount of materiel k accepted by the CDP
$D_{v}^{k}$	Amount of demand for material k at the DP	$Q_{l}^{k}$	Reserve at the CDP with respect to material k
$\chi_{o}$	Minimum demand rate	$E_{l}$	Throughput capacity of the CDP
$s_{ol}^{km}$	Number of means of transportation of $x_{ol}^{km}$	$s_{lv}^{km}$	Number of transportation means of $x_{lv}^{km}$
$s_{ol}^{m}$	Reserved transportation capacity from SP to CDP	$\mu_{\text{1}}$ and $\mu_{\text{2}}$	Different weight coefficients of the OF
$s_{lv}^{m}$	Reserved transportation capacity from CDP to DP	z	Optimal solution
Fit	Fitness function	V(t+1)	Ion update velocity
x(t+1), x(t)	Particle update position and particle update position	ε	Inertia factor
$g_{\text{1}}$, $g_{\text{2}}$	Particle movement acceleration	$r_{\text{1}}$, $r_{\text{2}}$	Uniformly distributed random numbers with values in the range of [0,1]
y(t), $y^{\prime }\left(t\right)$	HOP of a single particle and all particles of the population	f(x(t+1)), $f(x(t+1{)}^{!})$	Target values of left whisker and right whisker respectively

## Supporting information

S1 Dataset The minimal dataset required to reproduce the figures and results reported in this study is provided as Supporting Information (S1 Dataset). The dataset contains the original data used to generate the figures and experimental results presented in the manuscript.
(XLSX)
